# Interplay between *Eimeria acervulina* and *Cryptosporidium parvum* during In Vitro Infection of a Chicken Macrophage Cell Line (HD11)

**DOI:** 10.3390/life13061267

**Published:** 2023-05-27

**Authors:** Shahinaz Taha, Tran Nguyen-Ho-Bao, Lisa Maxi Berberich, Sandra Gawlowska, Arwid Daugschies, Zaida Rentería-Solís

**Affiliations:** 1 Institute of Parasitology, Centre for Infection Medicine, Faculty of Veterinary Medicine, Leipzig University, An den Tierkliniken 35, 04103 Leipzig, Germany; 2 Deparment of Preventive Medicine and Veterinary Public Health, Faculty of Veterinary Medicine, University of Khartoum, P.O. Box 32, Shambat 13314, Khartoum North, Sudan; 3 Faculty of Veterinary Medicine, College of Agriculture, Can Tho University, Can Tho 900000, Vietnam; 4 Albrecht-Daniel-Thaer Institute, Rudolf-Breitscheid-Str. 38, 04463 Größpösna, Germany

**Keywords:** *Eimeria acervulina*, poultry, macrophages, coccidia, *Cryptosporidium parvum*, innate immunity, in vitro

## Abstract

Background: *Eimeria acervulina* is a frequent intestinal pathogen of chickens, causing economic impact on the poultry industry. *Cryptosporidium parvum* is a neglected parasite in chickens. However, because of its zoonotic potential, poultry cryptosporidiosis may pose a risk to public health. Little is known about the parasite–host interactions during coinfection with both parasites. In this study, we investigated the possible interactions during in vitro coinfection of *E. acervulina* and *C. parvum* in a chicken macrophage cell line (HD11). Methods: HD11 cells were inoculated with *E. acervulina* and *C. parvum* sporozoites and incubated 2, 6, 12, 24, and 48 h post infection (hpi). Mono-infections for each parasite were also investigated. Real-time PCR was used to quantify parasite replication. Additionally, macrophage mRNA expression levels of IFN-γ, TNF-α, iNOS, and IL-10 were measured. Results: For both parasites, multiplication was, in most groups, lower in the coinfection group (COIG) compared with mono-infections. However, at 6 hpi, the number of *C. parvum* copies was higher in co-infections. Intracellular replication started to decrease from 12 hpi onward, and it was almost undetectable by 48 hpi in all groups. Infections resulted in low expression of all cytokines, except at 48 hpi. Conclusions: Infection of avian macrophages with both *E. acervulina* and *C. parvum* seemed to hinder intracellular replication for both parasites in comparison to mono-infection. A clear reduction in intracellular parasites from 12 hpi onward details the important role potentially played by macrophages in host control of these parasites.

## 1. Introduction

Chicken coccidiosis is an intestinal disease caused by the ubiquitous *Eimeria* genus [1] with significant economic importance for the poultry industry [2]. The disease can range from mild infection to acute enteritis with high mortality. *Eimeria* sp. infection can predispose to infection by other opportunistic pathogens, such as *Clostridium* spp., *Campylobacter* spp., or *Salmonella* spp. which can exacerbate the clinical condition of the host [3,4,5,6]. Seven *Eimeria* species are usually associated with chicken coccidiosis [1,7], among which *Eimeria* (*E*.) *acervulina* is one of the most common [7]. *E. acervulina* infects the upper part of the small intestine (duodenum), and the disease is mostly moderated. However, single *E. acervulina* infections are not the norm [6,7] and mixed infections with other *Eimeria* species or bacteria can be fatal [6].

*Cryptosporidium* (*C*.) *parvum* is a zoonotic apicomplexan parasite and a major cause of mild to severe gastrointestinal disease in humans and domestic animals [8,9,10]. It can infect the small intestine of a broad range of species [10,11,12,13], including birds. *C. parvum* infection in domestic fowl is not very common and occurs mostly as a subclinical condition [12,13,14]. In chicken, *Cryptosporidium* species predominantly invade the airways (respiratory cryptosporidiosis) and the bursa of Fabricius or cecum (intestinal cryptosporidiosis) [15,16,17]. Although seldom reported, classical small-intestine infections related to *C. parvum* as seen in mammals can occur in chickens [17,18], and infected birds can shed oocysts in the feces, which could pose a risk for human infection [12,13,19].

One of the first lines of defense against invading pathogens is the innate immune system. Along with heterophils and dendritic cells, macrophages play an imperative role in the inactivation and elimination of avian pathogens [20,21,22]. They achieve this through chemotaxis and phagocytosis, amongst other functions [22]. Additionally, they are uniquely situated at the interface between the innate and adaptative immune responses [22]. The latter is due in part to their antigen-presenting abilities [22]. During eimeriosis, the macrophages not only actively inactivate and destroy *Eimeria* spp. but also serve to transport sporozoites to the main regions of infection [23,24,25]. *C. parvum* utilizes mammal macrophages in a similar fashion [26]. Yet, the role of the avian macrophage has not been thoroughly explored in *Cryptosporidium* spp. infections in birds [27].

*C. parvum* and *E. acervulina* are both intracellular parasites that can infect the same site of the small intestine. Moreover, possible coinfections may impact not only parasite invasion and replication, but also, more importantly, the host immune response. The objective of this study was to evaluate possible interplay between both parasites in terms of intracellular replication and immune response activation during coinfection of *E. acervulina* and *C. parvum*.

## 2. Materials and Methods

### 2.1. Parasite Maintenance, Excystation, and Purification of Sporozoites

*E. acervulina* oocysts were kindly provided by MSD Animal Health Innovation GmbH (Schwabeheim, Germany). The oocysts were maintained through biannual passages in healthy 11 day old chicks, and sporulated oocysts were purified according to Eckert et al. [28] Purity of the isolate was assessed before and after each passage by multiple PCR assays for seven *Eimeria* spp. According to Andreopoulou et al. [7]. Finally, sporozoite excystation and purification were carried out following Rentería-Solís et al.’s [29] methodology.

*C. parvum* oocysts were routinely passaged in neonatal calves; oocyst purification was conducted according to Najdrowski et al. [30]. The *C. parvum* oocysts were from an in-house isolate belonging to the sub-genotypes IIaA14G1R1 and IIaA15G2R1 [31]. *C. parvum* sporozoite excystation was performed following the protocol of Berberich [32]. Briefly, *C. parvum* oocysts were pelleted at 10,000 rpm (9500× *g*) for 4 min at room temperature (RT) and then placed on ice. They were subsequently pretreated with 1 mL of 5.25% sodium hypochlorite (NaOCl) in cold PBS (1×) and incubated for 5 min on ice. The oocysts were then centrifuged at 10,000 rpm for 4 min at RT. After centrifugation, the supernatant was discarded, and the pellet was washed with PBS (1×) 2–3 times. Afterwards, the oocysts were resuspended in excystation medium consisting of 0.8% sodium taurocholate (Sigma, Steinheim, Germany) dissolved in RPMI 1640 culture medium. Oocysts were then pre-incubated at 15 °C for 1 h, and thereafter incubated at 37 °C with 5% CO_2_ for 1:30 h. The excystation rate and sporozoite counts were determined using a Neubauer counting chamber (Mediparts, Oberhausen, Germany) for both *E. tenella* and *C. parvum*.

### 2.2. Cell Culture

The chicken macrophage cell line HD11 is an immortalized line originated from chicken bone marrow [33]; the HD11 cells used in this study were kindly provided by the Bio Bank of the German Federal Research Institute for Animal Health, Insel Riems, Germany (Friedrich Löffler Institute, FLI). HD-11 cells were seeded in 24-well plates (5 × 10^4^ cells/well), cultured in RPMI-1640 medium, and supplemented with 8% fetal bovine serum (FBS), 2% chicken serum (CS), 100 IU penicillin, 100 µg/mL streptomycin (ThermoFisher Scientific, Dreieich, Germany), and 2.5 µg/mL amphotericin (Biochrom, Berlin, Germany). Cultures were maintained at 37 °C in 5% CO_2_ until confluent.

### 2.3. In Vitro Infection Assay

Confluent HD-11 monolayers (80% to 90% confluency) were infected with *C. parvum* (2 ×10^5^ sporozoites/well, MOI = 1) and *E. acervulina* (2 × 10^5^ sporozoites/well, MOI = 1), and then incubated at 41 °C in 5% CO_2_ for 2, 6, 12, 24, and 48 h. Infections were visualized under a phase-contrast microscope after each timepoint and every 24 h thereafter. Three groups of infection were set for each timepoint: single infection of *C. parvum* (CPIG), single infection of *E. acervulina* (EAIG), and coinfection with both *C. parvum* and *E. acervulina* (COIG). Additionally, every timepoint included a negative control group consisting of uninfected HD-11 monolayers. All reactions were performed in triplicate. For the 48 h groups, monolayers were washed once with sterile PBS, and fresh medium was added to the wells 24 h post infection (hpi). After each incubation period, cells were trypsinized and centrifuged, and the pellet was used for DNA or RNA purification.

### 2.4. Quantification of E. acervulina and C. parvum by Real-Time Quantitative PCR (qPCR)

DNA was purified from the pelleted cells using the DNeasy Blood and Tissue kit (Qiagen, Hilden, Germany) according to the manufacturer’s protocol. For quantification of *E. acervulina*, amplification of the SCAR marker gene was conducted as described by Taha et al. [34] using the primers published by Blake et al. [35] (Table 1). Furthermore, replication of *C. parvum* was estimated using a qPCR assay targeting the HSP70 gene [36]. All reactions were performed on a Bio-Rad CFX Connect Real-Time PCR Detection System (Bio-Rad, Feldkirchen, Germany). Standard curves were generated by serial dilutions of plasmid DNA as previously published [36,37].

### 2.5. Reverse-Transcriptase and Cytokine mRNA Quantification Using Real-Time PCR

Purification of RNA was performed using the RNeasy kit (Qiagen, Hilden, Germany) following the kit’s instructions. Purified total RNA was DNase-treated (DNase I, RNase-free, Thermo Scientific, Dreieich, Germany), and complementary DNA (cDNA) was synthesized using a RevertAid first-strand cDNA synthesis kit (Thermo Scientific, Dreieich, Germany) as described by the manufacturer. Relative quantification of the following chicken cytokines was performed using SYBR green-based real-time PCR assays as previously published [38,39]: interferon gamma (IFN-γ), inducible nitric oxide synthase (iNOS), interleukin 10 (IL-10), and tumor necrosis factor alpha (TNF-α) (see Table 1). Relative quantification of mRNA was performed using the 2^−ΔΔCt^ method and expressed as n-fold differences. Normalization of Ct values was conducted with primers for the housekeeping gene glyceraldehyde 3-phosphate dehydrogenase (GAPDH).

### 2.6. Statistical Analysis

Kolmogorov–Smirnov and Shapiro–Wilk normality tests were used to determine the normal distribution of data. A two-way ANOVA test was used for comparison between different timepoints and groups of infection. Differences were considered significant when the *p*-value was ≤0.05. All statistical analyses were performed using the GraphPad Prism 9.2 software (GraphPad, San Diego, CA, USA).

## 3. Results

### 3.1. Quantification of E. acervulina and C. parvum

**Figure 1:**
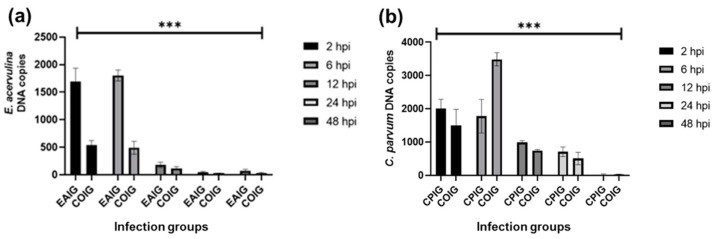
Quantification of intracellular parasites at different time points. (**a**) Quantification of *E. acervulina* DNA copies in single *E. acervulina* infection groups (EAIG) and coinfection (*E. acervulina* and *C. parvum*) groups (COIG). (**b**) Quantification of *C. parvum* DNA copies in single (CPIG) and coinfection (COIG) groups. Timepoints: 2, 6, 12, 24, and 48 hpi (order of the timepoint bars in the graphs, from left to right: 2 hpi, 6 hpi, 12 hpi, 24 hpi, and 48 hpi). *** *p* < 0.0001.

Monolayer integrity was visually assessed by microscopy at all timepoints. Overall, *E. acervulina* intracellular multiplication in the single groups (EAIG) was significantly higher (*p* < 0.0001) than in the coinfection groups (COIG) at all times (Figure 1a). Across the timepoints, *E. acervulina* copies in EAIG started notably higher than the COIG during the first 2 and 6 hpi. However, they significantly reduced their numbers from 12 hpi onward (24 and 48 hpi).

*C. parvum* multiplication between single and coinfection groups (CPIG and COIG, respectively) was not significant (*p* = 0.02381). Nevertheless, multiplication rates were higher in the CPIG than the COIG at all points except at 6 hpi (Figure 1b). Differences between time points, however, were significant for all groups (*p* < 0.0001) (Figure 1b). Parasite copies gradually decreased with time in the CPIG groups. In the COIG groups, however, the number of *C. parvum* copies notably increased at 6 hpi. Furthermore, from 12 hpi onward, *C. parvum* numbers in COIG were significantly reduced in comparison with CPIG (*p*-value < 0.0001).

### 3.2. Cytokine Analysis

**Figure 2:**
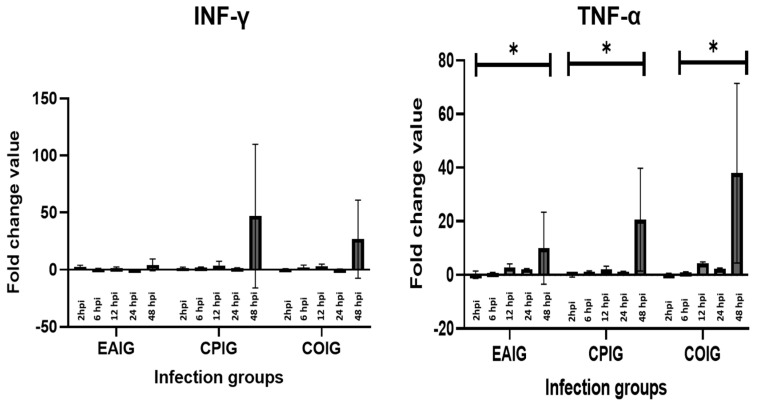
Modulation of the expression of INF-γ and TNF-α in HD11 cells during coinfection (COIG) and single infection (EAIG and CPIG), presented as fold changes of values recorded in uninfected controls at 2, 6, 12, 24, and 48 hpi (order from left to right); * *p* < 0.05.

The mRNA expression levels of four cytokines (Table 1) were measured in both single (EAIG and CPIG) and coinfection groups (COIG) at 2, 6, 12, 24, and 48 hpi by RT-qPCR (Figure 2). No statistically significant differences were found between single infection groups and coinfection groups for all cytokines (*p* > 0.05). However, there was a significant change across timepoints in the cases of TNF-α and IL-10 (*p* = 0.0334 and 0.0247, respectively).

**Figure 3:**
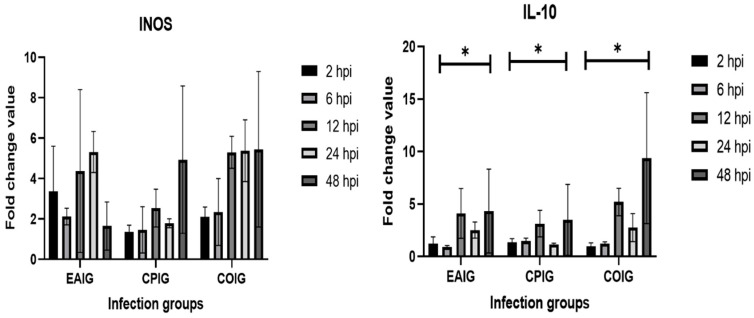
Modulation of the expression of INOS and IL-10 in HD11 cells during coinfection (COIG) and single infection (EAIG and CPIG), presented as fold changes of values recorded in uninfected controls at 2, 6, 12, 24, and 48 hpi (order of timepoint bars on the graphs, from left to right: 2 hpi, 6 hpi, 12 hpi, 24 hpi, and 48 hpi); * *p* < 0.05.

TNF-α expression only increased at 48 hpi. The mRNA expression levels in EAIG were lowest at 6 hpi for IFN-γ, TNF-α, and IL-10. Downregulation was solely detected for IFN-γ at 12 hpi and only in EAIG. Likewise, IFN-γ was downregulated in EAIG, as well as in COIG, after 24 hpi. At 48 hpi, all cytokine mRNA levels peaked in almost all infection groups, with the exception of iNOS in the EAIG group (Figure 3).

## 4. Discussion

We investigated the differences in parasite intracellular multiplication and host-cell cytokine expression between single and coinfections of *E. acervulina* and *C. parvum*. Dual infections of *Eimeria* spp. and *Cryptosporidium* spp. have been previously reported [18]; however, this is, to our knowledge, the first in vitro coinfection study in avian macrophages.

Zhang et al. [40,41] provided quantitative data during single and coinfection of *E. tenella* and *Toxoplasma gondii* in peripheral blood macrophages. *E. tenella* numbers were constantly reduced at all timepoints examined (2, 6, 12, and 24 hpi) in coinfected cultures [41]. Interestingly, the same authors measured the replication of *E. tenella* in poultry macrophages at 24, 48, and 72 hpi. In that study, *E. tenella* levels increased across timepoints, and detection of intracellular meronts was possible [40]. In our study, we did not investigate timepoints beyond 48 hpi. However, the number of parasites in our experiments was significantly reduced from 12 hpi onward. Michael [42] described the structural morphology of chicken macrophages 6 days after infection with *E. acervulina*. The author only found sporozoites within the macrophages [42]. It appears that *E. acervulina* simply fails to further develop into merozoites within macrophages, unlike *E. tenella*.

We detected a decrease in parasite DNA at 12 hpi. This could have been due to phagocytosis or removal of extracellular parasites after cell rupture during washing steps. During visual examination of monolayer integrity, we did not find noticeable damage to the cell structure. Other possible explanations for this discrepancy could be the different immunogenic potentials across *Eimeria* species [43], or the difference in MOI between studies. We used a lower MOI than Zhang et al., which may have impacted the macrophage response [40,41].

Zhang et al. [41] discovered that the phagocytic activity of the infected macrophages was hindered at 6 hpi for *E. tenella* infection but not for *T. gondii*. This could explain the peak in parasite replication we observed at 6 hpi in almost all cohorts of our study; the increment in intracellular *E. acervulina* at 6 hpi could have been the product of active cell invasion instead of phagocytosis. Moreover, the rapid decrease in parasite numbers after 6 hpi could indicate initiation of effective phagocytosis. However, macrophage phagocytosis was not investigated in our study.

*C. parvum* has the capacity to proliferate in mammal macrophages during in vitro infections [26,44]. Martinez et al. [26] were able to observe intracellular type I and II meronts of *C. parvum* in mouse macrophages at 24, 48, and 72 hpi. To the best of our current knowledge, no studies have previously been published that focused on intracellular mono- or coinfection dynamics of *Cryptosporidium* spp. in avian macrophages. In our study, coinfection with *C. parvum* and *E. acervulina* seemed to reduce the number of intracellular parasites for both species, except for *C. parvum* at 6 hpi in which coinfection showed a larger number of parasites than in the mono-infection. This was not the case for previous reports using *E. tenella* as a model, in which *E. tenella* showed a numeric advantage during coinfections of macrophages with *T. gondii* in comparison with single infections [40,41].

In addition to phagocytosis or their role as vehicles to infection sites [24,25,26,41], macrophages are also expert triggers of immune response, thanks in part to their ability to express cytokines [22]. For this study, we measured the expression levels of a cytokine involved in the inhibition of parasite replication in both *E. acervulina* and *C. parvum*: IFN-γ [42,43,44]. In our study, IFN-γ was only expressed at 48 hpi with very mild expression in the *E. acervulina* single infection, whereas the highest values were recorded in the coinfection and the *C. parvum* mono-infection. According to Dallout et al. [43], IFN-γ was not expressed in *E. acervulina*-infected macrophages during the first 48 hpi. In the present study, 48 hpi was also the timepoint with the lowest parasite replication, which could be explained by the possible role of IFN-γ in hindering *E. acervulina* and *C. parvum* intracellular multiplication. Additionally, IL-10 expression levels peaked at 48 hpi in all groups and were their lowest at 2 and 6 hpi. Given the regulatory activity of IL-10 against IFN-γ and previous data from *E. acervulina* in vivo infections [45], the low levels of IFN-γ mRNA in most groups could be explained by the continuous expression of IL-10 across infections and timepoints. iNOS was continuously expressed across all infection settings and timepoints. Interestingly, iNOS was continuously expressed with fold change values >1.5 for all groups and timepoints. Similar results were obtained in most groups for TNF-α, a known promoter of iNOS [46], which could explain the continuous presence of the latter, except at 2 hpi, when TNF-α expression was downregulated. These discrepancies could be further elucidated using proteomic methods. Furthermore, macrophage in vitro replication ability or cell viability during infection were not studied in this project but could be of importance for future investigations. This could help to elucidate possible hampering of macrophage function. Lastly, while in vitro models are an important tool for infection research, replication of our results in an in vivo model can be of significance, as applied in previous examples with other coinfections such as *Toxoplasma gondii* and *Eimeria tenella* [47].

## 5. Conclusions

Both *E. acervulina* and *C. parvum* gradually reduced their intracellular presence under the conditions of this study, which suggests an active role of macrophages in parasite control. Coinfection with both parasites seemed to affect their multiplication. We also replicated a previously reported inhibition in phagocytosis at 6 hpi for other apicomplexan parasites. Competition of different parasites for intracellular resources might play a role in parasite replication and should be further studied. Since coinfection with various species of the genus *Eimeria* and with other avian *Cryptosporidium* spp. may occur under natural conditions, coinfection studies deserve more attention.

## Figures and Tables

**Table 1:** Sequences of primers used in this study.

Oligonucleotide Identity	Primer Name, Primer Sequence (5′ to 3′)	Product Size (bp)	References
*E. acervulina* SCAR marker forward	Eac_qPCRf, CTC GCG TGT CAG CAC TAC AT	124	[35]
*E. acervulina* SCAR marker reverse	Eac_qPCRr, GAT AGC GTG CTT TGC CTT TC		[35]
*C. parvum* HSP70 forward	Cp_HSP70_f, AACTTTAGCTCCAGTTGAGAAAGTACTC	143	[36]
*C. parvum* HSP70 reverse	Cp_HSP70_r, CATGGCTCTTTACCGTTAAAGAATTCC		[36]
*C. parvum* HSP70 Taqman probe	HSP_70_SNA, AATACGTGTAGAACCACCAACCAATACAACATC		[36]
*Gallus domesticus* GAPDH forward	chicken_DAPDH_f, GGTGGTGCTAAGCGTGTTAT	264	[38]
*Gallus domesticus* GAPDH reverse	chicken_DAPDH_r, ACCTCTGTCATCTCTCCACA		[38]
*Gallus domesticus* IFN-γ forward	chicken_INF-γ_f, AGCTGACGGTGGACCTATTATT	259	[38]
*Gallus domesticus* IFN-γ reverse	chicken_INF-γ_r, GGCTTTGCGCTGGATTC		[38]
*Gallus domesticus* iNOS forward	chicken_iNOS_f, TGGGTGGAAGCCGAAATA	241	[38]
*Gallus domesticus* iNOS reverse	chicken_iNOS_r, GTACCAGCCGTTGAAAGGAC		[38]
*Gallus domesticus* IL-10 forward	chicken_IL-10_f, CGGGAGCTGAGGGTGAA	272	[38]
*Gallus domesticus* IL-10 reverse	chicken_IL-10_r, GTGAAGAAGCGGTGACAGC		[38]
*Gallus domesticus* TNF-α forward	chicken_TNF- α_f, CTTCTGAGGCATTTGGAAGC	380	[39]
*Gallus domesticus* TNF-α reverse	chicken_TNF- α_r, ACTGGGCGGTCATAGAACAG		[39]

## Data Availability

Not applicable.
